# Pseudovolvulus of the sigmoid colon after percutaneous endoscopic gastrostomy tube placement: A case report

**DOI:** 10.1016/j.ijscr.2020.02.049

**Published:** 2020-02-28

**Authors:** Motohiro Kikuawa, Akira Kuriyama, Hayaki Uchino

**Affiliations:** Emergency and Critical Care Center, Kurashiki Central Hospital, 1-1-1 Miwa, Kurashiki, Okayama, 710-8602, Japan

**Keywords:** PEG, percutaneous endoscopic gastrostomy, CT, computed tomography, NG, nasogastric, Percutaneous endoscopic gastrostomy, Sigmoid mesocolon, Hernia, Complications

## Abstract

**Introduction:**

Percutaneous endoscopic gastrostomy (PEG) provides long-term enteral nutritional access for patients with inability to eat. Although considered safe, PEG tube placement is associated with complications. We report a rare case of PEG-related sigmoid colon pseudovolvulus.

**Presentation of case:**

A 78-year-old man with a history of Parkinson’s disease developed severe abdominal pain and vomited continuously 50 days after PEG tube placement. Contrast-enhanced computed tomography revealed internal herniation of the sigmoid colon between the abdominal wall and the stomach at the gastrostomy site. Intraoperatively, the gastrostomy tube penetrated the sigmoid mesentery, which rotated around the tube, and the sigmoid colon was herniated towards the upper abdomen. The herniated colon was reduced and Hartmann’s procedure was performed. Subsequently, gastrostomy was reinforced with anterior gastropexy. The postoperative course was uneventful.

**Discussion:**

This case highlights the need for caution when placing a PEG tube because of a mobile sigmoid mesocolon, raising the awareness of potential major complications. Complications can be avoided by directly visualising the intraabdominal organs using laparoscopic gastrostomy or laparoscopic-assisted PEG. However, these methods require general anaesthesia. Thus, the presence of redundant colons should be determined in advance to assess the risk of sigmoid mesocolon perforation. We should also assess the patients’ swallowing function and estimate whether it may recover with rehabilitation before deciding to place a PEG tube.

**Conclusion:**

PEG tube should be considered after careful patient evaluation. If PEG is required, clinicians should recognise the patient-specific risks and consider other surgical procedures to avoid complications.

## Highlights

• Percutaneous endoscopic gastrostomy (EPG) is a safe procedure, but complications may occur.
• Major complications of PEG may present with volvulus-like conditions.
• The need for percutaneous endoscopic gastrostomy should be carefully evaluated.
• Laparoscopic gastrostomy and laparoscopic-assisted PEG have been proposed.
• Risk of complications should be timely recognized to enable proper treatment.

## 1 Introduction

Percutaneous endoscopic gastrostomy (PEG) is a modality of choice for providing long-term enteral nutritional access for patients with neurological disorders, such as stroke and Parkinson’s disease [1]. Although this procedure has been modified over time and is generally considered safe, PEG tube placement is associated with potential complications, such as injuries to the spleen, liver, and bowel [2]. Although the use of PEG has become restricted worldwide in terms of quality of life improvement, it is still required and instituted in the most aging nation, Japan [3].

Herein, we report a rare case of PEG-related pseudovolvulus of sigmoid colon requiring emergency surgery. The work has been reported in line with the SCARE criteria [4].

## 2 Presentation of case

A 78-year-old man with a history of stroke underwent emergency laparoscopic cholecystectomy at our hospital for gangrenous cholecystitis. During his hospital stay, he came to require a nasogastric (NG) tube for nutritional access because of exacerbated dysphagia. He was thereafter transferred to another hospital for further rehabilitation. Unfortunately, his swallowing function did not improve and he eventually underwent PEG 2 months after the first surgery. Gastric wall transillumination, focal finger invagination, and safe-track needle technique had been used as safety procedures during PEG. Computed tomography (CT) was not performed before the procedure.

**Fig. 1:**
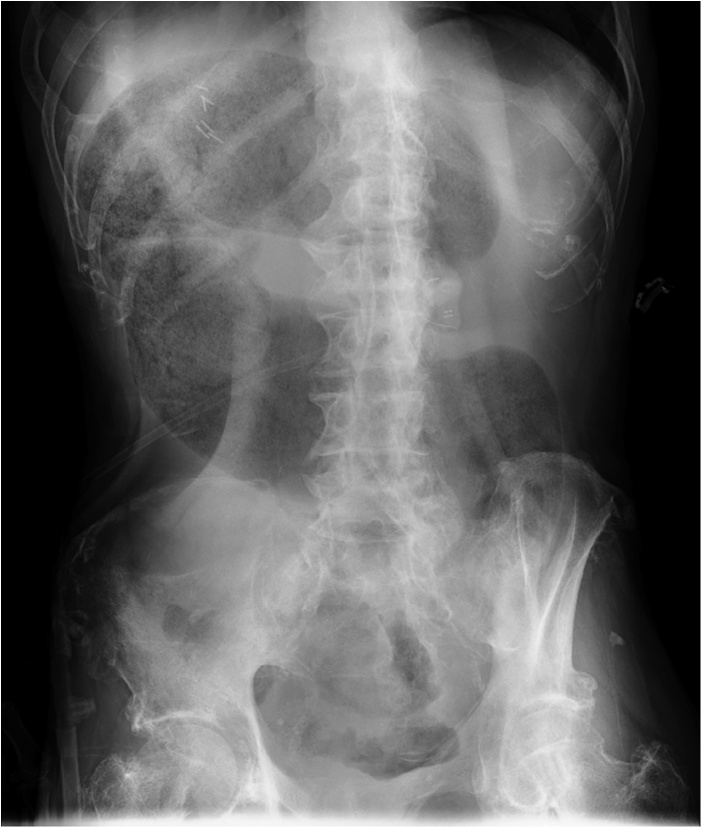
Distended sigmoid colon showing "coffee bean sign" on the abdominal X-ray.

**Fig. 2:**
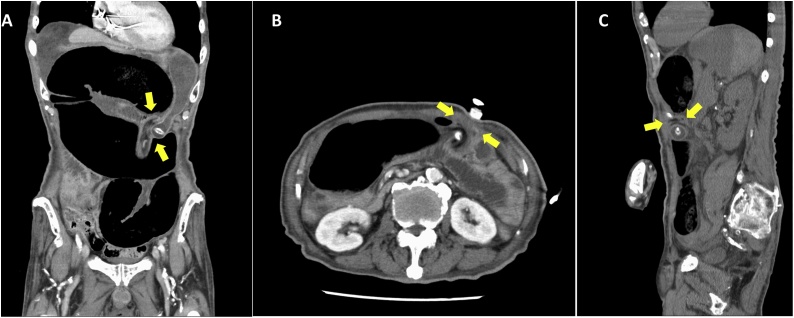
(A) Contrast-enhanced computed tomography revealed internal herniation of the sigmoid colon between the abdominal wall and the stomach as well as the whirling mesentery at the gastrostomy site on the coronal veiw, on the axial view (B), and on the sagittal view (C).

Fifty days after PEG tube placement, the patient developed severe abdominal pain and vomited continuously. He was referred to us for further treatment because of suspected small bowel obstruction on the CT scan. On arrival, his vital signs were stable. Abdominal distension with severe generalised tenderness was present. Laboratory analysis was remarkable only for leucocytosis (10.8 × 10^3^/μL) and abdominal X-ray showed “coffee bean sign”, indicating sigmoid colon volvulus (Fig. 1). Contrast-enhanced CT revealed internal herniation of the sigmoid colon between the abdominal wall and the stomach at the gastrostomy site with ischemic changes (Fig. 2). Because of the ischemic changes and the possibility that the PEG tube was related to the volvulus, endoscopic treatment was deemed impossible and emergency surgery was performed. Intraoperatively, we found that the PEG tube penetrated the sigmoid mesentery, which rotated around the tube, and the sigmoid colon was herniated towards the upper abdomen, resulting in a sigmoid volvulus-like condition. The herniated colon was reduced and Hartmann’s procedure was performed. Subsequently, the PEG tube was reinforced with anterior gastropexy. The patient had an uneventful postoperative course and was again transferred to the previous hospital for further rehabilitation.

## 3 Discussion

Gastrostomy is mainly indicated in patients with inability of oral food intake due to neuromuscular disorders or upper gastrointestinal tract obstructive lesions, such as cancer [1]. In Japan, long-term tube nutrition is also indicated in patients with dysphagia due to old age or stroke. Despite the worldwide campaign to discourage the gastrostomy use in such patients [5,6], it is still prevalent in Japan [3,7]. Therefore, knowledge of the procedure and management of patients with gastrostomy tubes is necessary.

PEG is broadly accepted as a safe guide for gastrostomy tube positioning. However, although uncommonly, complications may occur. Minor complications include superficial peristomal infection, leakage, granuloma, and tube occlusion or dislodgement [2]. Major complications include haemorrhage, peritonitis, internal organ injury, ileus, aspiration pneumonia, tumour seeding, and buried bumper syndrome [2,8,9]. Major complications are less frequent but more severe.

Few case reports have described major complications of PEG that presented with volvulus-like conditions. Walker et al. reported on a case of sigmoid intra-abdominal herniation and volvulus in a 42-year-old man with Parkinson’s disease [10]. The patient presented with acute abdominal pain, vomiting, and constipation 8 months after PEG tube placement. Laparotomy revealed sigmoid mesocolon perforation by the PEG tube and gastric outlet obstruction due to compression from the volvulus. The sigmoid colon was ischemic and Hartmann’s procedure was performed. Martins et al. described the case of a 47-year-old man with cerebral palsy who experienced fever, cough, and constipation 2 days after the PEG procedure [11]. Physical examination revealed signs of peritoneal irritation, and a PEG probe revealed dark drainage. Exploratory laparotomy revealed mesenteric torsion leading to small bowel volvulus, with no evidence of intestinal malrotation. The volvulus was successfully surgically untwisted. The patient tolerated diet via the gastrostomy tube. Roos et al. presented the case of a 39-year-old man with kyphoscoliosis that caused restrictive respiratory disease and advanced multiple sclerosis [12]. He developed abdominal distension, nausea, vomiting, and high gastric output from the PEG tube, indicating small bowel obstruction, 4 or 5 days after PEG tube placement. Laparotomy confirmed mid-jejunal obstruction. The PEG tube had traversed the small bowel mesentery twice, without damaging the bowel per se. One side of the PEG tube remained in the stomach, while the other side was threaded off the mesentery and replaced through a new incision in the skin. In our case, the sigmoid colon herniated toward the upper abdomen, while the gastrostomy tube penetrated the sigmoid mesentery, which rotated around the gastrostomy tube.

The patients in the aforementioned cases as well as ours had neurologic disorders. Patients with neurologic disorders are potentially at high risk of large bowel redundancy because chronic constipation may lead to colon dilatation and elongation [13]. The cause of constipation in these patients is multifactorial. First, these patients often have autonomic dysfunction or dysautonomia [14]. Second, the medications used for treatment of their neurologic disorders (e.g., dopamine agonists for Parkinson’s disease) are associated with constipation [15]. Third, long-term laxative use intended to treat constipation in such patients may damage the myenteric plexus, resulting in subsequent colonic dilatation [13]. Fourth, an inverse relationship has been found between physical activity and the risk of constipation, and most patients with neurologic disorders are hospitalized and bed-ridden [16]. Thus, the dilated and redundant sigmoid and its mesentery could freely move inside the abdominal cavity, and the PEG tube had incidentally perforated the mesentery.

As gastrostomy is placed mainly endoscopically, these cases highlight the need for caution of mobile colon and raise the awareness of potential major complications during PEG tube placement. These complications could be avoided by directly visualising the intraabdominal organs. Laparoscopic gastrostomy and laparoscopic-assisted PEG have been proposed. Kaya et al. described laparoscopic gastrostomy [17] and Thaker et al. reported a laparoscopic-assisted PEG procedure [18].

In laparoscopic gastrostomy, a purse-string suture is placed on the anterior stomach wall via an intraabdominal technique and is lifted by another thread pulling the suture. A hole is made at the suture centre on the anterior stomach wall using hook cautery and a Foley catheter is inserted. The advantage of laparoscopic gastrostomy is the good visualisation of the intraabdominal cavity, which lowers the risk of intraabdominal organ injury [17].

In laparoscopic-assisted PEG, which combines laparoscopic gastrostomy with PEG, one laparoscopic port is placed to inspect the intraabdominal cavity and upper gastrointestinal endoscopy is performed to determine the place of PEG tube insertion. These two procedures are performed simultaneously. Next, the PEG tube is placed via a standard technique used in transoral PEG. The merit of laparoscopic-assisted PEG is direct visualisation of both the intragastric and intraabdominal cavities, which enables identification of obstacles on the stomach lining, such as ulcers or cancer, and displaced structures between the stomach and the abdominal wall, such as the liver, small bowel, transverse or sigmoid colon, or the omentum [18].

Further, Gauderer et al. proposed a “hybrid” minimally invasive gastrostomy that uses laparotomy access along with endoscopic procedure [19].

All these methods, however, require general anaesthesia. Hence, the presence of redundant colon and the risk of sigmoid mesocolon perforation by the PEG tube should be determined in advance.

Physicians need to ask themselves the following fundamental questions: Do we really need PEG for this patient? Do we have any alternative routes of feeding this patient? The patient’s swallowing function should be evaluated to estimate whether it may recover with rehabilitation before deciding to place a PEG tube. Further, long-term PEG feeding is discouraged because it increases the risk of mortality in individuals with dementia and eating problems [20]. Recently, the concept of “comfort feeding only” has become an integral part of advanced care planning. Patients are offered food and water at each meal until they refuse or cannot feed further. For patients with low possibility of dysphagia recovery, particularly those with advanced dementia, advanced care planning should be discussed with them and their families in terms of their dignity and quality of life before their diseases progress. Thus, the need of tube feeding should be individually evaluated.

## 4 Conclusion

Although PEG is generally a safe procedure, complications may occur. In this case, the complication was due to a redundant colon and the lack of attention for a possible colon displacement at the PEG site before and during the procedure. The need for PEG in patients should be carefully evaluated and withholding or withdrawing feeding tubes be considered in terms of patients’ dignity and quality of life. In case of PEG placement, the risk of complications should be timely recognised, and if needed, another surgical approach should be considered.

## Sources of funding

This manuscript did not receive any specific grant from funding agencies in the public, commercial, or not-for-profit sectors.

## Ethical approval

As a case report without Protected Health Information, no ethics approval was required for this article.

## Consent

Written informed consent was obtained from the patient's family for the publication of this case report and any accompanying images. A copy of the written consent is available for review by the Editor-in-Chief of this journal.

## Author contribution

MK and AK contributed to patient care, performed the literature search, drafted the manuscript, and critically revised the manuscript. HU contributed to patient care and critically revised the manuscript.

## Registration of research studies

N/A.

## Guarantor

Akira Kuriyama.

## Provenance and peer review

Not commissioned, externally peer-reviewed.

## Declaration of Competing Interest

All authors have no conflicts of interest to declare.
